# A Sustainable Ambulance Operation Model in a Low-Resource Country (the Democratic Republic of Congo) 

**DOI:** 10.1155/2018/8701957

**Published:** 2018-08-28

**Authors:** Tae-Hun Lee, Jae-Hyun Han, Ashish Ranjan Sharma, Young-A Choi, Dong Won Kim, Sang-Soo Lee, Moo-Eob Ahn

**Affiliations:** ^1^Departments of Emergency Medicine, Hallym University-Chuncheon Sacred Heart Hospital, Chuncheon, Gangwon-Do, 24253, Republic of Korea; ^2^Gangwon Telemedicine Center, Hallym University College of Medicine, Chuncheon, Gangwon-Do, 24252, Republic of Korea; ^3^Institute for Skeletal Aging & Orthopedic Surgery, Hallym University-Chuncheon Sacred Heart Hospital, Chuncheon, 24252, Republic of Korea

## Abstract

Due to an increase in traffic collisions, the demand for prehospital medical services is on the rise, even in low-resource countries where emergency ambulance services have not been previously provided. To build a sustainable and continuous prehospital ambulance operation model, it is necessary to consider the medical system and economic conditions of the corresponding country. In an attempt to construct a prehospital ambulance operation model that ensures continuous operation, a pilot “emergency patient transporting service from field to hospital” operation was established for approximately three months in Kinshasa, the capital of the DR Congo. To construct a continuously operating model even after the pilot operation, willingness to pay (WTP) by type of emergency medical and transport service was investigated by implementing the contingent valuation method (CVM). Using CVM, the WTP for prehospital emergency services targeting ambulance services personnel, patients, policemen, and hospital staff participating in the pilot operation was calculated. The results of the pilot operation revealed that there were a total of 212 patients with a mean patient number of 2.4 per day. A total of 155 patients used the services for hospital transport, while 121 patients used the services for traffic collisions. Traffic collisions were the category in which ambulance services were most frequently needed (66.2%). Pay services were most frequently utilized in the home-visit services category (40.9%). Based on these results, eight independently operated ambulance operation models and sixteen models that utilize hospital medical personnel and policemen already belonging to existing institutions were proposed. In an effort to implement emergency medical ambulance services in the DR Congo, medical staff receiving pay for performance (incentive pay) should be deployed in the field and on call. Accordingly, with respect to sustainable development goals, various pay-for-service models should be used.

## 1. Introduction

Trauma comprises 12% of the world's burden of disease, with the number of trauma patients reaching nearly five million [1]. The 2010 world's burden of disease report noted that noninfectious disorders (such as trauma) and noncontagious diseases (such as AIDS, malaria, or tuberculosis) are significant causes of premature death [2]. Consequently, effective measures should be taken in low-resource countries to overcome the problem of inadequate domestic budgets and lack of operational experience. Furthermore, basic human rights, such as the “right to live” or “quality of life,” should also be ensured. This study suggests building an ambulance operation model that can even be sustained in a low-resource country by considering the existing medical system and economic status of the host country.

The aim of emergency medical service (EMS) systems is to provide immediate and proper treatment in emergency situations [3, 4]. EMS systems usually involve management and time dependent transportation service to render the needed care within a local community-based health care system, such as hospitals [5]. Furthermore, the ultimate goal is to reduce mortality and disability rates while at the same time promoting health. Nearly all countries operate a prehospital emergency service by utilizing their medical system and manpower. The representative prehospital emergency services include either the* Anglo-American* model, which utilizes paramedics as physician surrogates, or the* Franco-German* model, in which a physician physically rides in an ambulance [6]. However, after benchmarking the prehospital emergency service models from other countries, each country should develop its own unique prehospital emergency service system to fit its own medical and social circumstances [7]. The prehospital emergency services have recently evolved, centering on the issue of how ambulance services are operated. The construction of prehospital emergency service models in various countries was not related to the increase in traffic collisions, which paved the way for building more improved prehospital emergency ambulance models [8]. A rapid increase in traffic collisions led to the 1973 legislation of the emergency medical services system (EMSS) ACT in the United States [5]. The increased number of deaths following a rise in traffic collisions and inappropriate emergency treatments gave birth to the 1964 enactment of “the laws and regulations on emergency hospitals” in Japan and the Republic of Korea. In 1995, the “the Emergency Medical Services Act” legislation triggered the commencement of the construction of the prehospital emergency services in full-scale. It has been reported that there are 1,200,000 traffic deaths in the world every year. Of these deaths, young people between the ages of 10 to 24 years old comprise the largest proportion. Ninety percent (90%) of all the deaths occur in developing or underdeveloped countries. These countries (low-resource countries) collectively share 54% of all the cars in the world, which incurs a 1% to 1.5% reduction of the gross national product (GNP) [9]. Low-resource countries require a sustainable ambulance operation model that can offer in-the-field emergency treatment and prompt transportation of traffic collisions patients to an appropriate hospital.

The present study was conducted in Kinshasa, the capital of the DRC, where there is no operational prehospital emergency service model. The ten-year long civil war in DRC resulted in an extremely deteriorated healthcare infrastructure. Due to the disproportionate cost of medical care facilities relative to income levels, the poor have been largely alienated from the benefits of health care services. In particular, the recent investments by China have vigorously expanded road construction-related infrastructure in Kinshasa, resulting in an acute increase in traffic collisions. Furthermore, the prehospital emergency services are not yet operational and appropriate emergency patient care has not been performed due to the absence of emergency medicine specialists in the hospital EMS [10]. According to the WHO-Global Status Report on Road Safety [9], the number of motor vehicles registered in the DRC as of the year 2010 is approximately 350,000 and is on the rise till date. The rate of fatalities from traffic collisions was very high, at 79%. Furthermore, approximately 70 to 80% of severe traffic collisions patients would die prematurely without proper care due to lack of transportation facilities to a hospital and the lack of proper ambulance system. In an effort to build a sustainable ambulance operation model, in collaboration with the DRC Ministry of Public Health, our research team subsidized ambulances, emergency treatment equipment for the ambulances, and education for operational personnel.

## 2. Methods

### 2.1. Pilot Service

Pilot ambulance services from the field of injury to a hospital were conducted in certain regions of Kinshasa, the capital of DRC, for three months from September to December of 2013. Prior to the pilot service, we subsidized a total of twelve ambulances (equipped with nineteen medical devices) and educated 320 individuals (such as physicians, nurses, drivers, and administrative personnel) on basic life support (BLS) and advanced trauma life support (ATLS) for a total of six sessions. We also provided simulated dispatch training twice. The EMS team, affiliated with the Division of Emergency Medical and Disaster Assistance of the Ministry of Health and Welfare, was organized and commissioned to operate and manage ambulances. The team performed the pilot operation for three months from September 18, 2013, to December 15, 2013.

### 2.2. Survey Questionnaire on the Issues of Ambulance Service Needs, Ranking of Preferential Areas, and Willingness to Pay

Four versions of questionnaires were prepared for each category of the targeted patients, emergency ambulance team, hospital personnel, and policemen. Each version was drawn up to include three areas of investigation. First, the questions included sex, age, educational level, monthly income, predicted transportation time from the field to a hospital, and patient's status at the site of the traffic collisions for the investigation of demographic characteristics of the responders. Second, participants were asked to rank areas with the highest need for ambulance services. The questions also included a selection for preferred services, such as (1) transportation service from the field to a hospital, (2) hospital-to-hospital transfer, and (3) home-visit medical service for the verification of user intention toward “ambulance service for pay.” A cross-analysis among the groups was used to verify intergroup differences. Third, to build a model for a sustainable ambulance service operation, WTP for prehospital emergency services was computed using the CVM method on based on surveys performed on the patients, emergency ambulance personnel, policemen, and hospital staff. After WTP for the three prehospital emergency services types, ANOVA or the Kruskal-Wallis test was used to verify the WTP differences among these groups.

### 2.3. Modeling for a Sustainable Ambulance Service Operation

Hypothetically, to build an ambulance service model, there were a total of twenty-four operative scenarios, depending on the principal operators (emergency ambulance team, hospitals, or the police organization), manpower (physician and paramedic personnel), and an external supporting structure. Here, we calculated the total revenue from the sum of WTP for all three types of services with the expenses incurred by fixed costs (vehicle expenses and labor costs) and variable costs (fuel costs and supplies). The results of the breakeven point (BEP) for each of the twenty-four operative scenarios were also computed and compared. The mean number of operations per day and the average expenses for each operation were computed for BEP with functional formulas:

(1) The functional formula for calculating the number of prehospital emergency operations per day, the amount charged for each type of prehospital emergency service, and the total revenue earned from the number of ambulances operated for one year (365 days).

(2) The functional formula for calculating the vehicle, facilities, and equipment expenses to operate one ambulance, the labor costs for operating an ambulance (physician, nurse, and driver), the fuel costs for a single emergency ambulance operation, medical supplies costs and vehicle maintenance expenses for each operation, telephone operator labor costs for working in the operation control center, and other yearly expenses.

(3) The functional formula for calculating the BEP for total revenues and expenditures for one year and the number of ambulance operations that were allowed from the minimum of zero to a maximum of 24 a day.

### 2.4. Equations

1. The total revenue for one year (US$) = 365×z×(∑xiyi)
2. The total expenditure for one year (US$) = [(Expenses for ambulance, facilities and equipment/duration of utilization) + 12(months) × (the labor costs for a physician × the number of physicians + the labor costs for a nurse × the number of nurses + the labor cost for a driver × the number of drivers) + 365 × (fuel costs+ vehicle maintenance costs + supplies costs) ∑xi]z + 12 months × labor cost for a telephone operator × the number of telephone operators + e (error cost) = [10,000+12(1,000 × 3+500 × 3+250 × 3) + 365 × (1.58 + 3.68) ∑xi]z + 12 × 250 × 6+e
3. BEP (The total revenues for one year–the total expenditures for one year ≧ 0) ⇒ 0 ≦ 365×z×∑xiyi - [10,000+63,000+365 × 5.26∑xi]z+18,000 + e

### 2.5. Decision Variables

  xi is the number of operations per day by type of emergency ambulance service.
  yi is the amount charged per operation by type of emergency ambulance service.
  z is the number of ambulances.

Ambulance, facilities, and equipment's depreciation cost for 10 years is $100,000/year; labor costs based on three shifts a day for each ambulance are as follows: for a physician: US$1,000/month; a nurse: $500/month; a driver: $250/month; a telephone operator at the operation control center (three shifts a day, two telephone lines): $250/month. Variable costs are as follows: fuel costs: $1.58/operation; medical supplies and vehicle maintenance costs: $3.68/operation; and other expenses are not calculated.

### 2.6. Limiting Factors

  0 ≤ ∑xi ≤ 24
  0 < z (whole number)

## 3. Results

### 3.1. Status of Ambulance Utilization during the Pilot Operation

During the pilot operation, there were 162 telephonic reports. Of these, hospital transport incidents comprised 155 (95.7%), and cases involving traffic collisions were 121 (74.7%). The total number of patients was 212 or an average of 2.4 cases per day.

### 3.2. Circumstances Requiring Ambulance Transportation Service and Pay-for-Service Areas

The highest ambulance need was for traffic collisions with a weighted frequency of 308 points. This was followed by 191 points for nontraffic injuries, 189 points for emergency disease, 84 points for hospital-to-hospital transport, and 25 points for drug intoxication (Table 1).

The responders replied that the areas where pay-for-service could be implemented were home-visit service (40.9%), field-to-hospital transportation service (32.3%), and hospital-to-hospital transportation service (26.8%), in decreasing order. In particular, the area of home-visit medical service had the highest proportions among the targeted service personnel—patients (75%), policemen (50%), and hospital staff (38.1%). On the other hand, the responders answered that the field-to-hospital transportation service had the highest potential for pay-for-service in the emergency ambulance personnel category (35.7%). The results of the analysis show that the differences among targeted service personnel were statistically significant (p=0.011 with a p value less than 0.05 considered statistically significant (Table 2).

### 3.3. Comparative Analysis of WTP for the Types of Transportation Services

The WTP comparative analysis results by type of ambulance service among targeted service personnel are shown in Table 3. The mean WTP for the field-to-hospital transportation service for patients was $26.63, which was the lowest. Meanwhile, the value for hospital staff was $60.40, which was the highest amount. Next, the average WTP for the hospital-to-hospital transportation service for patients was $31.53, the lowest amount. The hospital staff was the highest, at $76.83. Lastly, the mean WTP for the home-visit medical service for patients was $5.32, the lowest figure, while the value for emergency service personnel was the highest, at $86.77.

### 3.4. BEP Calculations for Prehospital Emergency Ambulance Service Operation Models by Model Type

The lowest expenses, i.e., US$32.15 and US$52.24, were computed for scenario 2 and 4 models, respectively. These models are physician-included independent models with three types of services, consisting of either three-member (a physician, a nurse, and a driver) or two-member (a physician and a driver) crews. The scenario 14 and 16 models had the lowest expenses at US$51.68 and US$52.24, respectively (Table 4). These models are physician-included models that take advantage of an existing hospital organization and provide three types of services. These models consisted of either three-member (a physician, nurse, and driver) or two-member (a physician and driver) crews. The scenario 22 and 24 models, which utilize an existing police organization, had the lowest expenses at US$5.51 and US$5.88, respectively. The scenario 22 model provides three types of services, while the scenario 24 model provides two types of services. These two emergency medical technician (EMT) models consisted of either three personnel (two nurses and a driver) or two personnel (a nurse and a driver) crews.

## 4. Discussion

This study attempts to develop a sustainable ambulance operation model that would be appropriate for the current socioeconomic and political circumstances of a low-resource country. For this study, a pilot operation was conducted in certain parts of Kinshasa, the capital of the Democratic Republic of Congo (DRC). Various EMSS operations, including ambulance operations, have been attempted in low-resource countries, but such efforts are largely performed with foreign models. Nevertheless, it is difficult to succeed in any public development assistance program without a sustainable operation model and without an adequate understanding of the social and political circumstances of a low-resource country [11, 12]. Along with benchmarking of the successful EMSS of advanced countries, the uniqueness of the medical environment that exists in each country should be considered. Prehospital emergency services may be affected by the history of each country, regional characteristics, socioeconomic/cultural background, scientific technologies, and the method through which health and safety services are provided [13, 14].

As a national policy goal, the DRC has established a plan to introduce a sustainable emergency ambulance system so that all of its citizens will be able to access healthcare services at a reasonable price. Further, the operation aims to promote healthy conditions by providing citizens with high quality, universal, and continuous healthcare services regardless of social status, location, policy, or religion by participating in regional communities [15]. The budget execution rate in the public health sector of DRC has increased every year from 0.17% of the total governmental budget in 2000 to 5.93% in 2007. Nevertheless, due to civil war, 90% of the public health budget has been supported by donor countries, and only 3.91% of the actual budget was executed. The high out-of-pocket health expenditure has shown the limitations of the public health sector [15, 16]. Funding for uninterrupted operation is necessary to continuously operate an ambulance service. Continuous foreign subsidies or building a profitable operational model is necessary due to the inability to procure funds for operations with the governmental budget in low-resource countries. Numerous countries with an EMSS ambulance service are operating various ambulance services for pay. In the Republic of Korea, emergency patients are able to receive a firefighter ambulance services at no cost and air ambulance from the field to a hospital. However, ambulance services from the private sector provide hospital-to-hospital or hospital-to-home transportation at cost [17]. There are differences in charges for BLS and advanced life support (ALS) ambulances at US$25.00 and US$62.00, respectively, for a distance of 10 km or less, with an additional charge of 0.83 Korean won or US$1.00 for every additional 1 km. In the United States, each state has a unique prehospital emergency system, but most systems are pay-for-services and are covered by Medicare or private insurance plans. In Los Angeles, BLS ambulance transportation costs US$1,000-US$1,100 and ALS transportation costs US$1,200-US$1,300 on average.

In this study, we made the assumption that there would be three pay-for service types as follows: (1) field-to-hospital transportation, (2) hospital-to-hospital transportation, and (3) home-visit medical service. This investigation attempted to suggest a sustainable emergency ambulance service model based on these assumptions. First, in an effort to establish the need (intent of utilization) for these three service types, a questionnaire survey was conducted on experienced patients, emergency ambulance personnel, and hospital staff as part of a pilot service. The intent to utilize user-pay services was confirmed for the three types in decreasing order as follows: home-visit medical service, 40.9% (52 personnel); field-to-hospital transport, 32.3% (41 personnel); and hospital-to-hospital transport, 26.8% (34 personnel). In deciding charges for each service type, the CVM was applied to the experienced users for the appraisal in each service. In particular, the so-called contingent valuation method has been put forward as a way to determine the willingness to pay for various goods and services. This is done by setting up a surrogate contingent market for nonmarket resources (goods) through a questionnaire. In principle, CVM is a useful method for eliciting passive use (existence) values in cases where utilization and consumption of resources are not clearly delineated [18]. Patients, emergency service personnel, hospital staff, and policemen who had experience using the service were interviewed 1:1 to establish WTP for ambulance service. Thus, the social value and the potential for user-pay service were analyzed by ambulance service types. Based on these findings, we attempted to coordinate principal operators of ambulance services (independent organization, existing hospital, or police organization), medical crew members (physician, paramedics), and three types of revenue-earning models to minimize user burden and at the same time create a sustainable ambulance operation model. Many different types of CVM question formats (deduction method) may include “open-ended,” “bidding,” and “payment-card” questions. In consideration of the difficult local circumstances in conducting the preliminary survey and primary investigation in this study, the “open-ended” CVM method and direct open-ended questioning without suggesting any amount was used to obtain WTP. The mean WTP for “field-to-hospital transportation service" was US$26.63; policemen, US$37.00; hospital staff, US$60.40; and ambulance service personnel, US$54.50. The mean WTP for “hospital-to-hospital transport service” was US$31.53; policemen, US$49.50; hospital staff, US$76.83; and ambulance service personnel, US$66.61. The mean WTP for “home-visit medical service” was US$5.32; policemen, US$35.10; hospital staff, US$60.63; and ambulance service personnel, US$86.77.

Principal emergency service operator, types of crew/manpower, and the crew numbers determine an ambulance operational model [19]. Regarding principal emergency service operators, an independent operator as well as an existing hospital or police organization may be considered. The ambulance crew may consist of a physician or paramedics. A physician crew can provide facilities for all the three prehospital emergency services (field-to-hospital, hospital-to-hospital, and home-visit medical service), but a paramedics-only crew cannot provide a home-visit medical service. Thus, independent medical emergency operation permits eight models, while as many as sixteen models are possible if an existing hospital or a police organization is used. Of these 24 models, a sustainable ambulance operation model that minimizes the expenditure for the field-to-hospital transportation service and has a high public interest would consist of a two-physician-crew model utilizing a hospital organization. It is estimated that this model would earn the highest revenue of US$52.24 per service. If the revenue were supplemented with subsidies from public aid or governmental funding for vehicle and variable expenses, the estimated maximum revenue would be as much as US$67.33.

In the Republic of Korea, paramedics are utilized for the ambulance crew. In Seoul, one operation control center and 144 prehospital emergency service teams are on call. A team of 129 emergency ambulance personnel, 3 physicians, and 6 telephone operators are stationed in the operation control center, while 1,297 ambulance personnel are actively engaged in 144 emergency ambulance teams. According to 2014 prehospital emergency ambulance dispatch cases (484,494), the cost for a single dispatch was approximately US$135.00 after reflecting the annual income for crews, vehicle maintenance fee, supply expenses, fuel costs, and other expenditures.

In the DRC utilizing an existing hospital organization, a two-physician crew ambulance, providing field-to-hospital and hospital-to-hospital transport services, as well as home-visit medical service, can earn a revenue of US$52.24 per service, and the revenue can pay for the ambulance operation costs. With the implementation of such a model, an ambulance service in the DRC can be provided at approximately 39% of the ambulance operation costs in the Republic of Korea. The functional formula for this operation model is generated to build a sustainable ambulance operation model for the field-to-hospital transport service at the prehospital stage in a low-resource country. The equation is set up to find a way to minimize user charges for field-to-hospital ambulance services. The fuel cost, supply expenses, and vehicle maintenance cost for a single emergency ambulance service, regardless of travel distance or equally shared workloads, are a linear functional formula. The linear attribute of the functional formula together with constructing a scenario model estimated only with the data from the prehospital stage is the limitations. Another limitation is that the results were obtained from a pilot operation, which was only conducted in a limited region during a predetermined period.

In conclusion, in order to maintain the ambulance operating system in DRC, which is relatively rich in manpower resources but lacks hardware resources and operating funds, the fields-to-hospital transport service operating at low-cost for public purpose is quite feasible while hospital-to-hospital and home visiting service with the an affordable cost for public could be alternate models.

## Figures and Tables

**Table 1:** Requirements for ambulance services (units: frequency, %).

Areas	1^st^ place	2^nd^ place	3^rd^ place	Weighted total frequencies ^×^
Traffic collisions	88 (66.2)	15 (11.3)	14 (10.5)	308
Non-traffic injuries	23 (17.3)	46 (34.6)	30 (22.6)	191
Emergency disease	14 (10.5)	49 (36.8)	49 (36.8)	189
Drug intoxication	2 (1.5)	5 (3.8)	9 (6.8)	25
Hospital to hospital transporting	6 (4.5)	18 (13.5)	30 (22.6)	84
Others	-	-	1 (0.8)	1

^×^Weighted total frequencies (score) = frequencies of 1^st^ place cases × 3 + frequencies of 2^nd^ place cases × 2 + frequencies of 3^rd^ place cases × 1.

**Table 2:** Emergency ambulance services costs (units: frequency, %).

Targeted service personnel (group)	Field-to-hospital transporting service	Hospital-to-hospital transporting service	Home-visiting-medical service	Total	p-value
Patients	3(18.8)	1(6.3)	12(75.0)	16(100)	0.011
Policemen	8(40.0)	2(10.0)	10(50.0)	20(100)	0.011
Hospital staff	20(31.7)	19(20.2)	24(38.1)	63(100)	0.011
Ambulance personnel	10(35.7)	12(42.9)	6(21.4)	28(100)	0.011
Total	41(32.3)	34(26.8)	52(40.9)	127(100)	0.011

^×^p<0.05, ^××^p<0.01.

Data are Frequency (rate) P-values are for Χ^2^ as appropriate.

**Table 3:** The estimated costs for emergency ambulance services (Units: frequency, USD).

Item	Group	N	Mean	SD	F- or *χ*^2^	p-value
Field-to-hospital ambulance service	Patients	19	26.63	20.13	21.537	0.001^××^
	Policemen	20	37.00	33.73		
	Hospital staff	63	60.40	31.00		
	Ambulance personnel	30	54.50	27.05		

Hospital-to-hospital ambulance service	Patients	19	31.53	24.80	34.703	0.001^××^
	Policemen	20	49.50	25.64		
	Hospital staff	63	76.83	23.27		
	Ambulance personnel	31	66.61	32.80		

Home-visiting medical service	Patients	19	5.32	2.96	73.213	0.001^××^
	Policemen	20	35.10	32.95		
	Hospital staff	63	60.63	12.30		
	Ambulance personnel	31	86.77	21.66		

^×^p<0.05, ^××^p<0.001.

Data are frequency (rate) and the mean± S.D. P values are for Χ^2^, t test as appropriate.

**Table 4:** Analysis of prehospital emergency medical services based cost-service (units: USD).

Principal operator	personnel composition	Scenario	Service type	Daily op. rate	Op. costs
Independent Emergency service team Operation-[Operation Control Center Manpower]	3-crew physician model (physician + nurse + driver)	1	field-to-hospital	100%	22.27
		2	field-to-hospital	50%	-32.15^*∗*^
			Hospital-to-hospital	25%	66.61
			Home-visiting	25%	86.77
	2-crew physician model (physician + driver)	3	field-to-hospital	100%	18.16
		4	field-to-hospital	50%	-40.37
			Hospital-to-hospital	25%	66.61
			Home-visiting	25%	86.77
	3-crew EMT model (nurse + nurse + driver)	5	field-to-hospital	100%	18.16
		6	field-to-hospital	75%	2.01
			Hospital-to-hospital	25%	66.61
	2-crew EMT model (nurse + driver)	7	field-to-hospital	100%	14.05
		8	field-to-hospital	75%	-3.47
			Hospital-to-hospital	25%	66.61

Hospital organization utilized	3-crew physician model (physician+nurse+driver)	9	field-to-hospital	100%	21.93
		10	field-to-hospital	50%	-24.88
			Hospital-to-hospital	25%	76.83
			Home-visiting	25%	60.63
	2-crew physician model (physician+driver)	11	field-to-hospital	100%	17.82
		12	field-to-hospital	50%	-33.10
			Hospital-to-hospital	25%	76.83
			Home-visiting	25%	60.63
	3-crew physician incentive model (physician + nurse + driver)	13	field-to-hospital	100%	8.52
		14	field-to-hospital	50%	-51.68
			Hospital-to-hospital	25%	76.83
			Home-visiting	25%	60.63
	2-crew physician incentive model (physician + driver)	15	field-to-hospital	100%	8.24
		16	field-to-hospital	50%	-52.24
			Hospital-to-hospital	25%	76.83
			Home-visiting	25%	60.63

Police organization utilized	3-crew EMT model (nurse + nurse + driver)	17	field-to-hospital	100%	17.82
		18	field-to-hospital	75%	7.26
			Hospital-to-hospital	25%	49.50
	2-crew EMT model (nurse + driver)	19	field-to-hospital	100%	13.71
		20	field-to-hospital	75%	1.78
			Hospital-to-hospital	25%	49.50
	3-crew EMT incentive model (nurse + nurse + driver)	21	field-to-hospital	100%	8.24
		22	field-to-hospital	75%	-5.51
			Hospital-to-hospital	25%	49.50
	2-crew EMT incentive model (nurse + driver)	23	field-to-hospital	100%	7.96
		24	field-to-hospital	75%	-5.88
			Hospital-to-hospital	25%	49.50

*∗* denotes an income and Op. denotes operational cost.

## Data Availability

Data availability is provided within the article.
